# Synergistic cycles of protease activity and inflammation via PPARγ degradation in chronic obstructive pulmonary disease

**DOI:** 10.1038/s12276-021-00626-7

**Published:** 2021-05-21

**Authors:** Nakwon Kwak, Kyoung-Hee Lee, Jisu Woo, Jiyeon Kim, Chang-Hoon Lee, Chul-Gyu Yoo

**Affiliations:** 1 Division of Pulmonary and Critical Care Medicine, Department of Internal Medicine, Seoul National University Hospital, Seoul, South Korea; 2 Department of Internal Medicine, Seoul National University College of Medicine, Seoul, South Korea

**Keywords:** Inflammation, Cytokines

## Abstract

Inflammation, oxidative stress, and protease–antiprotease imbalance have been suggested to be a pathogenic triad in chronic obstructive pulmonary disease (COPD). However, it is not clear how proteases interact with components of inflammatory pathways. Therefore, this study aimed to evaluate the effect of neutrophil elastase (NE) on lipopolysaccharide (LPS)-induced interleukin 8 (IL-8) production and determine the molecular mechanism in human bronchial epithelial cells (HBECs). Immortalized bronchial epithelial cells and primary HBECs were used to investigate the impact of NE on LPS-induced IL-8 production. The molecular mechanism by which NE modulated LPS-induced IL-8 production was confirmed in elastase-treated C57BL/6 mice and primary HBECs obtained from COPD patients and healthy controls. The results showed that NE treatment synergistically augmented LPS-induced IL-8 production in both immortalized bronchial epithelial cells and primary HBECs. NE partially degraded peroxisome proliferator-activated receptor gamma (PPARγ), which is known to regulate IL-8 production in the nucleus. Treatment with a PPARγ agonist and overexpression of PPARγ reversed the NE-induced synergistic increase in LPS-induced IL-8 production. Moreover, PPARγ levels were lower in lung homogenates and lung epithelial cells from elastase-treated mice than in those from saline-treated mice. In accordance with the findings in mice, PPARγ levels were lower in primary HBECs from COPD patients than in those from healthy never-smokers or healthy smokers. In conclusion, a vicious cycle of mutual augmentation of protease activity and inflammation resulting from PPARγ degradation plays a role in the pathogenesis of COPD.

## Lung disease: getting to the root of inflammation

Tracing how a protein-degrading enzyme called neutrophil elastase (NE) interacts with cytokines, molecules that trigger inflammation, has revealed a potential new treatment target for chronic obstructive pulmonary disease (COPD). COPD is a progressive disease in which chronic inflammation damages lung function. Although it was known that cytokines play key roles in COPD, the detailed molecular mechanism was not well understood. Chul-Gyu Yoo at Seoul National University College of Medicine in South Korea and co-workers investigated the role that NE plays in inflammation in COPD. They found that NE degraded peroxisome proliferator-activated receptor gamma (PPARγ), a transcription factor that keeps cytokine production in check. Patients with COPD showed low levels of PPARγ, and boosting PPARγ or suppressing NE in cultured cells showed potential for reducing inflammation. These results highlight PPARγ as a candidate for development of COPD treatments.

## Introduction

Chronic obstructive pulmonary disease (COPD) is a progressive inflammatory disease characterized by persistent respiratory symptoms and airflow limitations^1^. The pathogenesis of COPD is complex, and inflammation, oxidative stress, and protease–antiprotease imbalance have been suggested to be a pathogenic triad in COPD^2^. Within this triad, the most well-known type of pathogenesis associated with COPD is chronic inflammation caused by exposure to noxious irritants, including cigarette smoke (CS). The inhalation of CS activates pattern-recognition receptors, including toll-like receptors (TLRs), stimulating an innate immune response that leads to the recruitment of neutrophils and macrophages to the lungs^3^ and activates airway epithelial cells^3^. Activation of these cells triggers the release of various inflammatory cytokines, such as interleukin 8 (IL-8), which acts as a chemoattractant for neutrophils; these recruited neutrophils perpetuate chronic inflammation in COPD^4,5^.

Chronic inflammation in COPD can persist even after smoking cessation^6^. The long-term effects of CS include airway structural damage that leads to secondary bacterial infection in the airways^6^. This bacterial colonization persistently provides antigens that induce airway inflammation. For example, lipopolysaccharide (LPS), which is produced in response to infection, activates airway epithelial cells to release IL-8^7^ and the resultant neutrophilic inflammation contributes to the development of COPD^8^.

In addition to chronic inflammation, oxidative stress caused by reactive oxygen species (ROS) contributes to the pathogenesis of COPD. The most well-known source of ROS in the pathogenesis of COPD is CS, which causes tissue destruction and DNA damage^9^. Moreover, CS reinforces oxidative stress by triggering the generation of additional ROS, further augmenting inflammation^2,10^. In addition to the crosstalk that occurs between the inflammatory processes and oxidative stress pathways, inflammation and protease enzymatic activities are also likely to be mutually reinforcing. On the one hand, inflammatory cells, especially neutrophils, secrete various proteases, including neutrophil elastase (NE), proteinase 3, myeloperoxidase, and cathepsin. On the other, NE increases the recruitment of neutrophils in response to the presence of chemotactic fragments that are produced by the cleavage of proteins in the extracellular matrix and from the destruction of lung tissue^11,12^. Moreover, we have previously reported that crosstalk occurs between the catalytic processes of NE and those leading to IL-8 production after exposure to CS^13,14^. Therefore, inflammation, oxidative stress, and protease–antiprotease imbalance constantly interact with each other to drive disease progression.

In contrast to CS, chronic inflammation caused by bacterial infection and its interaction with proteolytic activity remain unclear. In fact, IL-8, neutrophils, and NE activity are further increased in the bronchoalveolar lavage fluid of COPD patients with persistent airway bacterial infections^8^. However, the crosstalk between proteases and inflammation induced by bacterial infection has not been elucidated. Therefore, in this study, the aim was to evaluate the effect of NE on LPS-induced inflammatory responses and to determine the molecular mechanism in bronchial epithelial cells.

## Materials and methods

### Study subjects and animals

For animal experiments, female 6-week-old C57BL/6 wild-type (WT) mice were purchased from KOATECH (Pyeongtaek, South Korea). All animal experiments were approved by the Institutional Animal Care and Use Committee (No. 19-0220-S1A1[1]) of Seoul National University Hospital, Seoul, South Korea.

Normal human bronchial epithelial cells (HBECs) (BEAS-2B cells) were maintained in defined keratinocyte serum-free medium (Gibco by Life Technologies, Grand Island, NY, USA) at 37 °C and 5% CO_2_. Primary HBECs were provided by participants who underwent bronchoscopies for diagnostic or therapeutic purposes. These cells were obtained from the lobar and subsegmental bronchial lumens by bronchial brushing. All patients consented to providing their epithelial cells, and the Institutional Review Board of Seoul National University Hospital approved the study protocol (IRB No. H-1602-108-742).

### Reagents

Human sputum NE was acquired from Elastin Product Company (Owensville, MO, USA). The NE was dissolved in a solution containing 50% 0.02 M NaOAc (pH 5) and 50% glycerol. LPS, phenylmethylsulfonyl fluoride (PMSF), and rosiglitazone were obtained from Sigma-Aldrich (St. Louis, MO, USA). The selective elastase inhibitor Elaspol was purchased from Ono Pharmaceutical Co., Ltd. (Osaka, Japan). The anti-IκBα antibody was obtained from Cell Signaling Technology (Danvers, MA, USA). Anti-peroxisome proliferator-activated receptor gamma (PPARγ), anti-TLR4, anti-p65, anti-NE, and anti-glyceraldehyde 3-phosphate dehydrogenase (GAPDH) antibodies were all purchased from Santa Cruz Biotechnology (Santa Cruz, CA, USA).

### Protein extraction and western blot analysis

Total cellular proteins were extracted using 1× cell lysis buffer (Cell Signaling Technology). Membrane proteins were isolated using a membrane protein extraction kit (Thermo Fisher Scientific, Waltham, MA, USA). Protein concentrations were determined using the Bradford protein assay according to the manufacturer’s instructions (Bio-Rad, Hercules, CA, USA). Proteins were separated by 4–12% sodium dodecyl sulfate-polyacrylamide gel electrophoresis (SDS-PAGE) and then transferred to nitrocellulose membranes. The membranes were blocked with 5% skim milk blocking buffer for 1 h, followed by overnight incubation at 4 °C with primary antibodies in blocking buffer. The membranes were washed in washing buffer three times, followed by incubation with secondary antibodies for 1 h. After successive washes, the membranes were developed using a SuperSignal West Pico Chemiluminescent kit (Thermo Fisher Scientific).

### Flow cytometry

Cells were incubated with anti-TLR4 phycoerythrin (PE) or anti-immunoglobulin (Ig) G PE in 200 µL of incubation buffer for 45 min. Unreacted PE-TLR4 antibodies were removed. Cell-associated PE-conjugated antibodies were analyzed by flow cytometry using a FACSCalibur or a FACSCanto flow cytometer (BD Biosciences, San Jose, CA, USA).

### Quantification of IL-8 secretion

The levels of IL-8 in cell culture supernatants were measured using a commercially available human IL-8/CXCL8 DuoSet enzyme-linked immunosorbent assay (ELISA) kit (R&D System, Minneapolis, MN, USA) according to the manufacturer’s instructions.

### Transfection of plasmid vectors

Transfections of the control vector and the pAdTrack-CMV-PPARγ expression vector were performed using a Neon Transfection System (Thermo Fisher Scientific) according to the manufacturer’s instructions.

### Real-time quantitative polymerase chain reaction (RT-qPCR)

Total RNA was isolated using an RNeasy kit (Qiagen, Hilden, Germany). Total RNA (1 μg) was reverse-transcribed to cDNA using a reverse transcription system (Promega, Madison, WI, USA). Power SYBR Green PCR Master Mix (Applied Biosystems, Foster City, CA, USA) was used for amplification. The primers used in the study were as follows: IL-8 (fwd: 5′-GCA GCT CTG TGT GAA GGT GC-3′, rev: 5′-TCT GCA CCC AGT TTT CCT TG-3′); PPARγ (fwd: 5′-TGT GGG GAT AAA GCA TCA GGC-3′, rev: 5′-CCG GCA GTT AAG ATC ACA CCT AT-3′); TLR4 (fwd: 5′-CTG CAA TGG ATC AAA GGA CCA-3′, rev: 5′-TTA TCT GAA GGT GTT GCA CAT TCC-3′); and GAPDH (fwd: 5′-GAA GGT GAA GGT CGG AGT C-3′, rev: 5′-GAA GAT GGT GAT GGG ATT TC-3′).

### Immunofluorescent labeling

Cells were grown in 35 mm dishes, fixed in methanol for 10 min and washed three times. The cells were then incubated with the anti-p65 antibody diluted 1:100 in 3% bovine serum albumin (BSA) for 24 h. The cells were subsequently incubated with an Alexa Fluor 555 antibody (Thermo Fisher Scientific) diluted 1:100 in 3% BSA for 30 min. After successive washes, the cells were analyzed using EVOS digital color fluorescence microscopy (Thermo Fisher Scientific).

### PPARγ fragmentation and quantification

Cell lysates were incubated with either the vehicle control (50% glycerol, 50% 0.02 M NaOAc, pH 5) or the NE at 37 °C for 30 min. The reaction was terminated by adding an excess of western blot sample buffer. The samples were subjected to western blot analysis to quantify the concentrations of PPARγ and NE.

### Intratracheal administration of elastase

C57BL/6 WT mice were anesthetized, and saline or elastase was administered intratracheally (0.5 U in 100 μL of saline). The mice were sacrificed 1 day after elastase administration, and the lungs were isolated for immunohistochemical labeling.

### Immunohistochemistry

Lung tissues were fixed, embedded, sectioned at a thickness of 4 µm, and placed on slides for labeling using the Discovery XT automated immunohistochemistry staining system (Ventana Medical Systems, Inc., Tucson, AZ, USA). Tissue sections were deparaffinized and rehydrated. For antigen retrieval, a Cell Conditioning 1 (Ventana Medical Systems) standard (pH 8.4 buffer containing tris/borate/EDTA) was used. The sections were incubated with the anti-PPARγ antibody at 37 °C for 32 min, washed, and incubated with a secondary antibody for 20 min. After successive washes, the slides were incubated with 3,3-diaminobenzidine H_2_O_2_ substrate at 37 °C for 8 min, followed by counterstaining with hematoxylin and a bluing reagent. Stained cells were observed under a microscope (EVOS XL Core Cell Imaging System, Thermo Fisher Scientific).

### Statistical analysis

The data were analyzed with a two-tailed unpaired *t-*test or Mann-Whitney *U* test to assess significant differences between groups. All statistical analyses were performed using GraphPad Prism software (San Diego, CA, USA). A *P*-value < 0.05 was considered to be statistically significant.

## Results

### NE synergistically augments LPS-induced IL-8 production in lung epithelial cells

**Fig. 1:**
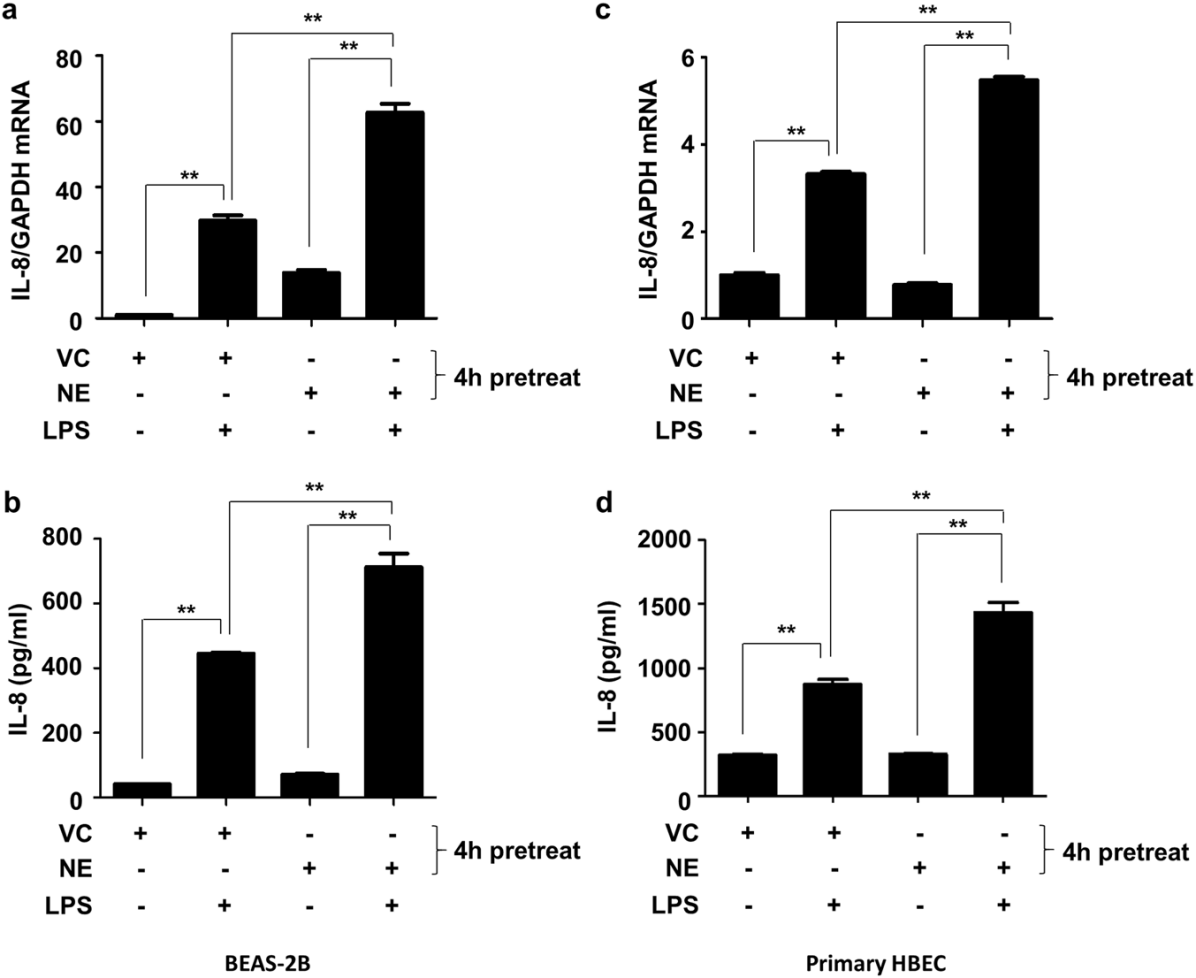
NE synergistically augments the LPS-induced production of IL-8 in lung epithelial cells. BEAS-2B cells (**a**, **b**) and primary HBECs (**c**, **d**) were pretreated with VC or NE (1 U/mL) for 4 h and then stimulated with LPS (1 µg/mL) for 16 h in the presence or absence of VC or NE. Total RNA was isolated from BEAS-2B cells (**a**) and HBECs (**c**), and quantitative real-time PCR analysis of IL-8 and GAPDH was performed. The levels of IL-8 in the supernatants of BEAS-2B cells (**b**) and HBECs (**d**) were measured by ELISA. The data represent mean ± SD; ^**^P < 0.05. Abbreviations: NE, neutrophil elastase; LPS, lipopolysaccharide; IL-8, interleukin 8; HBECs, human bronchial epithelial cells; VC, vehicle control; RNA, ribonucleic acid; PCR, polymerase chain reaction; GAPDH, glyceraldehyde 3-phosphate dehydrogenase; ELISA, enzyme-linked immunosorbent assays; SD, standard deviation.

The effect of NE on LPS-induced IL-8 production was assessed. As expected, stimulation with LPS (1 μg/mL) increased the number of IL-8 mRNA transcripts and protein concentrations in BEAS-2B cells and primary HBECs (Fig. 1). While treatment with NE alone did not affect the level of IL-8, pretreatment with NE for 4 h augmented the LPS-induced production of IL-8 in both BEAS-2B cells and primary HBECs (Fig. 1).

### Neither the TLR4 nor the IκB/NF-κB pathway is involved in NE-mediated augmentation of LPS-induced IL-8 production

**Fig. 2:**
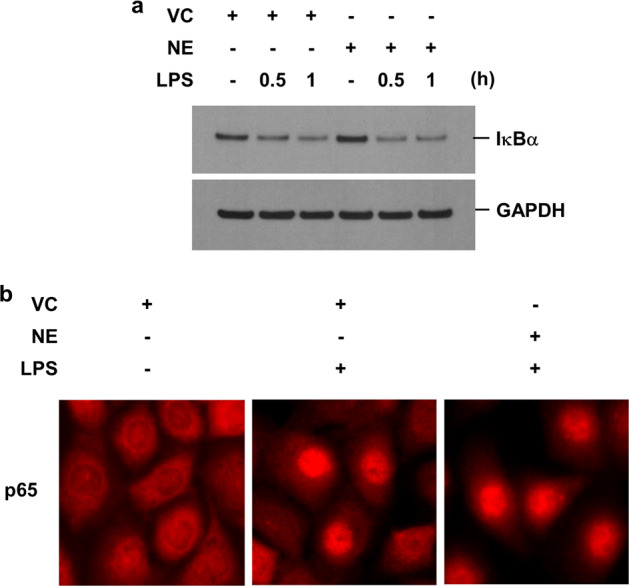
NE does not affect LPS-induced degradation of IκBα or the subsequent nuclear translocation of the NF-κB subunit p65. **a** BEAS-2B cells were pretreated with VC or NE (1 U/mL) for 4 h and then stimulated with LPS (1 μg/mL) for the indicated times in the presence or absence of VC or NE. Total cellular extracts were subjected to western blot analysis for IκBα and GAPDH. **b** Cells were pretreated with either VC or NE and then incubated with LPS for 1 h in the presence or absence of VC or NE. The cells were fixed and permeabilized for 10 min. Immunofluorescent staining of p65 was performed using an anti-p65 primary antibody, followed by an Alexa Fluor 555 secondary antibody. Cells were analyzed using EVOS digital color fluorescence microscopy. Abbreviations: NE, neutrophil elastase; LPS, lipopolysaccharide; IκBα, nuclear factor of kappa light polypeptide gene enhancer in B-cells inhibitor alpha; NF-κB, nuclear factor kappa B; VC, vehicle control; GAPDH, glyceraldehyde 3-phosphate dehydrogenase.

Since it is well known that LPS first binds to TLR4, which then activates the IκB/NF-κB pathway to ultimately result in IL-8 production^15^, whether the TLR4 or ΙκB/NF-κB pathway was involved in mediating the NE-mediated synergistic increase in LPS-induced IL-8 production was tested. TLR4 expression or its affinity for LPS should be increased if TLR4 is involved in mediating the synergistic increase in LPS-induced IL-8 production. However, while NE treatment increased the mRNA levels of TLR4 in BEAS-2B cells and HBECs (Supplementary Fig. 1a, b), it decreased the protein concentration of TLR4 in both the total cellular extracts and membrane fractions (Supplementary Fig. 1c–f). LPS stimulation led to IκBα degradation and the subsequent nuclear translocation of p65 (an NF-κB subunit); this outcome was not affected by NE treatment (Fig. 2a, b). IκBα phosphorylation was also not affected by NE treatment (data not shown). Thus, the TLR4 and IκB/NF-κB pathways are unlikely to be involved in mediating the NE-mediated synergistic increase in LPS-induced IL-8 production.

### NE causes the degradation of PPARγ

**Fig. 3:**
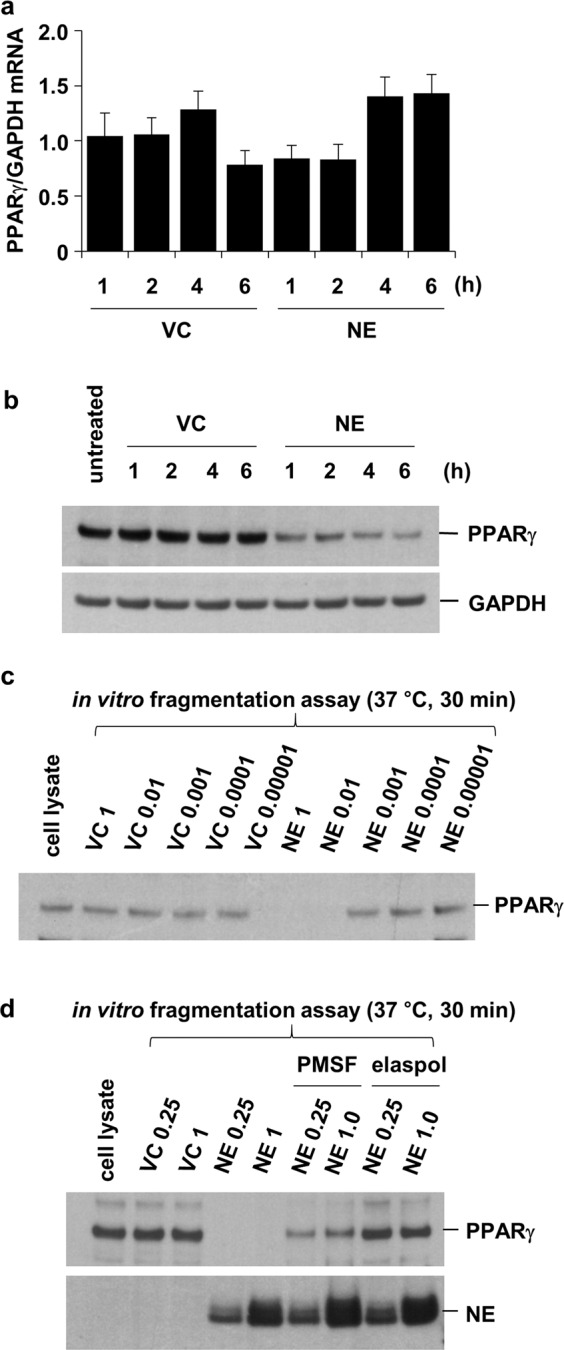
NE downregulates PPARγ through proteolytic degradation. BEAS-2B cells were treated with VC or NE (1 U/mL) for the indicated times. **a** Total RNA was isolated, and quantitative real-time PCR analysis of PPARγ and GAPDH was performed. The data represent mean ± SD. **b** Total cellular extracts were subjected to western blot analysis for PPARγ and GAPDH protein quantification. **c**, **d** Cell lysates were incubated with VC or NE (0.00001, 0.0001, 0.001, 0.01, 0.25, and 1 U/mL) in the presence or absence of a serine protease inhibitor (PMSF, 1 mM) or an elastase inhibitor (Elaspol, 100 μg/mL) at 37 °C for 30 min. The reaction was terminated by adding an excess of sample buffer; subsequently, the samples were subjected to western blot analysis for PPARγ quantification. Abbreviations: NE, neutrophil elastase; PPARγ, peroxisome proliferator-activated receptor gamma; VC, vehicle control; RNA, ribonucleic acid; PCR, polymerase chain reaction; GAPDH, glyceraldehyde 3-phosphate dehydrogenase; SD, standard deviation; PMSF, phenylmethylsulfonyl fluoride.

PPARγ, a ligand-activated transcription factor in the nucleus, has been reported to suppress the transcriptional activity of NF-κB, leading to a decrease in IL-8 production^16^. While the level of PPARγ mRNA was not affected by NE treatment in BEAS-2B cells (Fig. 3a), the concentration of PPARγ protein decreased (Fig. 3b, c). Coincubation with either a serine protease inhibitor (PMSF) or an elastase inhibitor (Elaspol) blocked the NE-induced decrease in PPARγ concentration (Fig. 3d). Thus, NE downregulates PPARγ through proteolytic degradation.

### PPARγ downregulation is responsible for the NE-mediated synergistic increase in LPS-induced IL-8 production

**Fig. 4:**
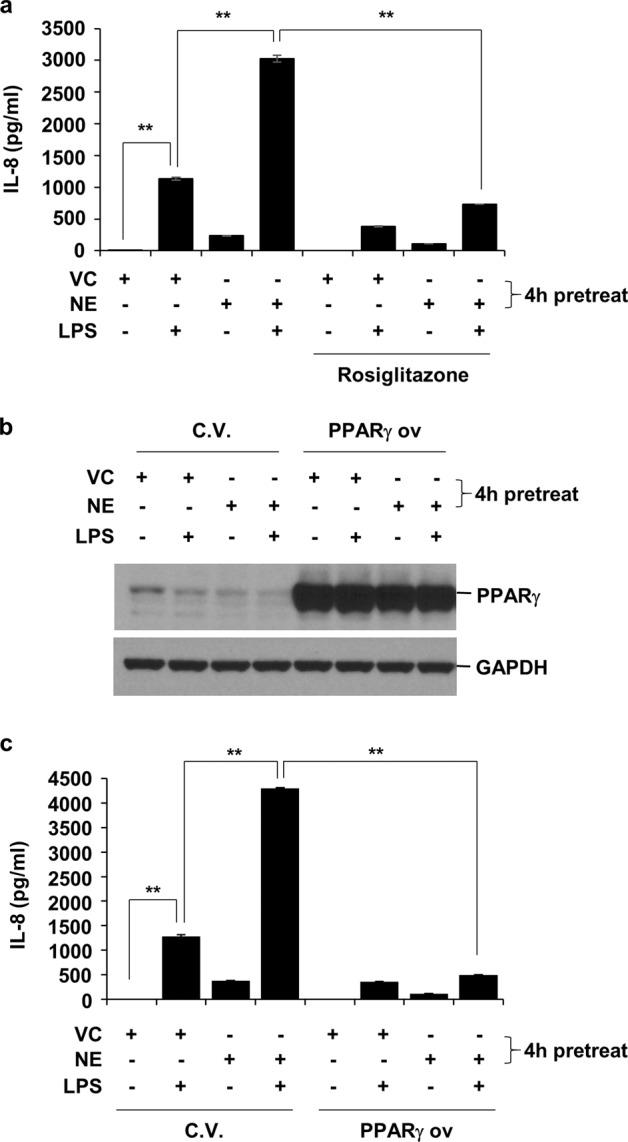
Treatment with either a PPARγ agonist or overexpression of PPARγ suppresses the NE-induced increase in IL-8 production in LPS-treated cells. **a** BEAS-2B cells were pretreated with rosiglitazone (50 μM) for 1 h, followed by VC or NE (1 U/mL) for 4 h in the presence or absence of rosiglitazone. The cells were subsequently stimulated with LPS (1 μg/mL) for 24 h in the presence or absence of rosiglitazone. **b**, **c** BEAS-2B cells were transiently transfected with a pAdTrack-CMV-PPARγ expression vector or a control vector. Forty hours after transfection, the cells were pretreated with VC or NE (1 U/mL) for 4 h, followed by stimulation with LPS (1 μg/mL) for 16 h in the presence or absence of VC or NE. The concentrations of IL-8 in the supernatants were determined by ELISA (**a**, **c**). The data represent mean ± SD; ^**^P < 0.05. Total cellular extracts were subjected to western blot analysis for PPARγ and GAPDH quantification (**b**). Abbreviations: PPARγ, peroxisome proliferator-activated receptor gamma; NE, neutrophil elastase; IL-8, interleukin 8; LPS, lipopolysaccharide; VC, vehicle control; ELISA, enzyme-linked immunosorbent assay; SD, standard deviation.

Next, the role of NE-mediated PPARγ downregulation in mediating the synergistic increase in LPS-induced IL-8 production was assessed. Stimulation of PPARγ by rosiglitazone suppressed the NE-mediated synergistic increase in LPS-induced IL-8 production (Fig. 4a). Moreover, the overexpression of PPARγ, which is not degraded by NE, also suppressed the NE-mediated synergistic increase in LPS-induced IL-8 production (Fig. 4b, c). These data indicate that PPARγ downregulation by NE is responsible for the synergistic increase in LPS-induced IL-8 production.

### PPARγ expression levels are decreased in airway epithelial cells in both elastase-treated mice and in patients with COPD

**Fig. 5:**
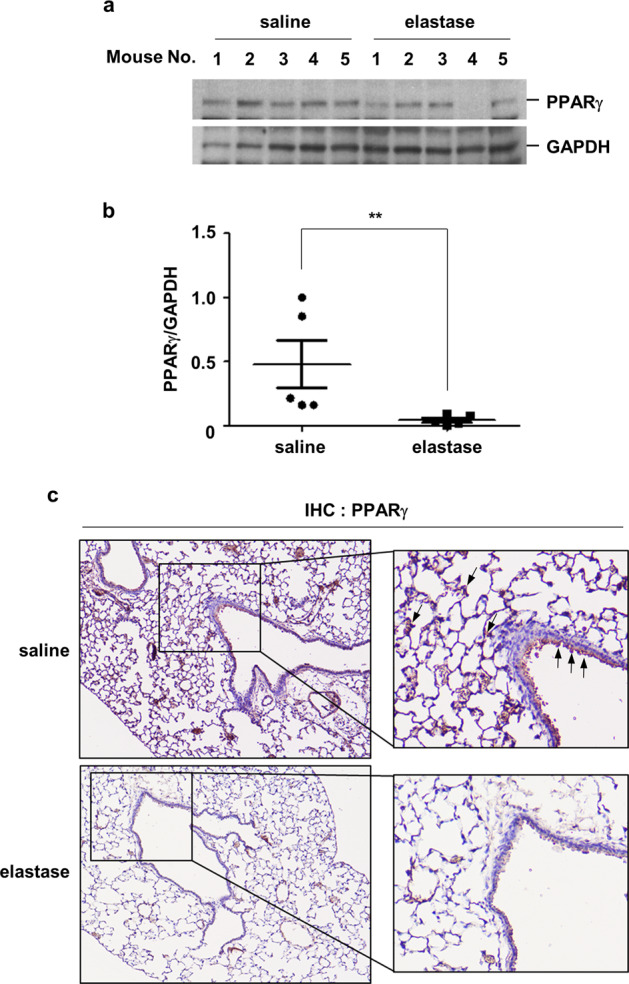
The expression levels of PPARγ are decreased in the lung epithelial cells of elastase-treated mice. **a**–**c** C57BL/6 mice were intratracheally administered either saline or elastase (0.5 U in 100 μL saline). The mice (*n* = 5 per group) were sacrificed 1 day after elastase treatment, and the lungs were isolated. Total lung protein lysates from the mice were subjected to western blot analysis for PPARγ and GAPDH quantification (**a**). The gel data were quantified using Scion image densitometry (**b**). The data represent mean ± SE; ^**^P < 0.05. **c** PPARγ immunohistochemical analysis in lung tissues collected from mice. Arrows indicate epithelial cells stained with PPARγ. Abbreviations: PPARγ, peroxisome proliferator-activated receptor gamma; GAPDH, glyceraldehyde 3-phosphate dehydrogenase; SE, standard error.

**Fig. 6:**
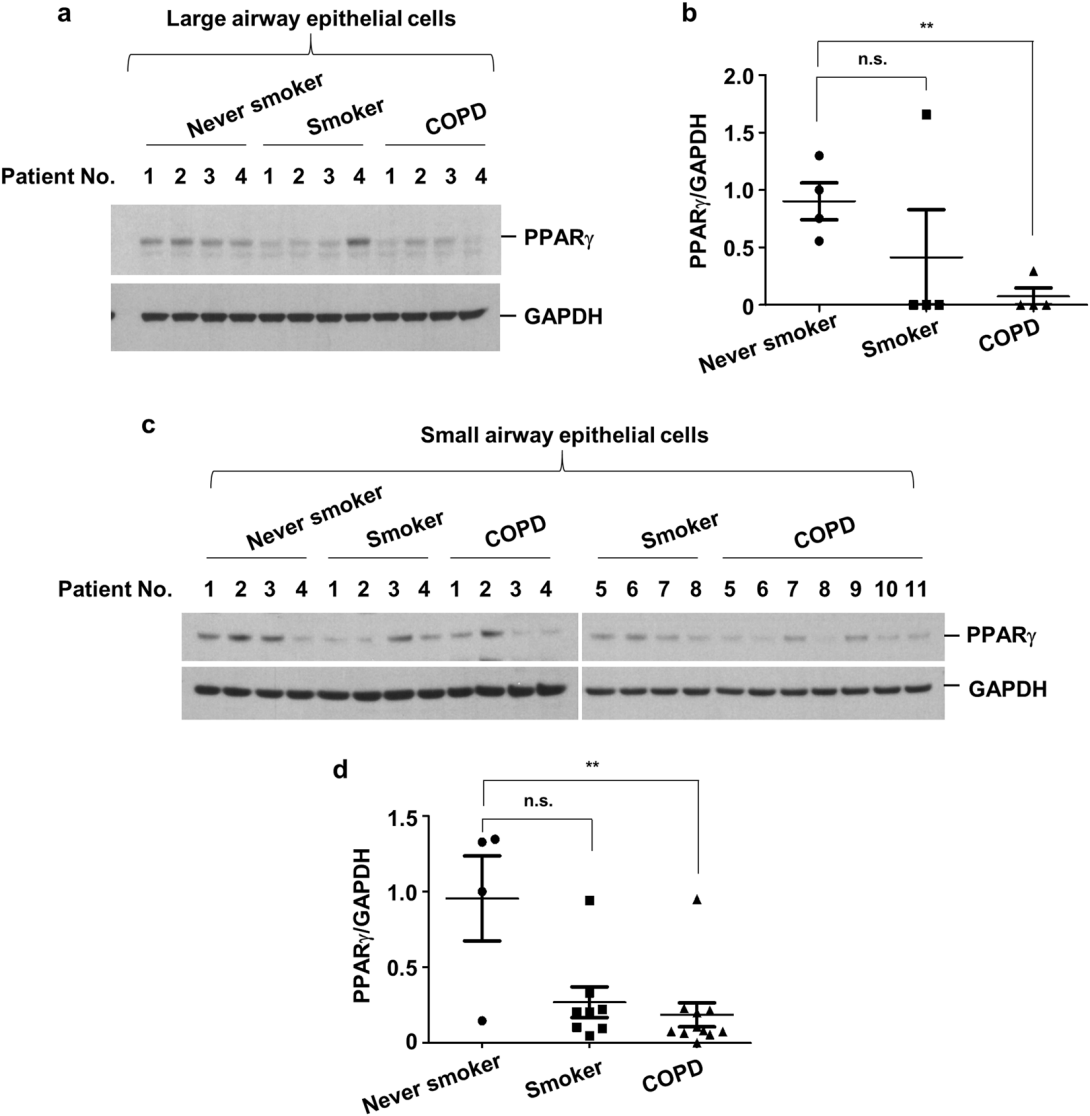
PPARγ levels are decreased in the airway epithelial cells of COPD patients. **a**, **b** Large airway epithelial cells were obtained from never-smokers (*n* = 4), smokers (*n* = 4) and COPD patients (*n* = 4). **c**, **d** Small airway epithelial cells were acquired from never-smokers (*n* = 4), smokers (*n* = 8) and COPD patients (*n* = 11). Total cell lysates were subjected to western blot analysis for PPARγ and GAPDH quantification (**a**, **c**). The gel data were quantified using Scion image densitometry (**b**, **d**). The data represent mean ± SE; ^**^P < 0.05. Abbreviations: PPARγ, peroxisome proliferator-activated receptor gamma; COPD, chronic obstructive pulmonary disease; GAPDH; glyceraldehyde 3-phosphate dehydrogenase; SE, standard error.

Finally, we evaluated whether these in vitro findings could also be observed in vivo. PPARγ expression levels were lower in lung homogenates from elastase-treated mice than in those from saline-treated mice (Fig. 5a, b). Immunohistochemical staining showed that the decrease in PPARγ expression was prominent in lung epithelial cells (Fig. 5c). In accordance with the findings in mice, the expression of PPARγ in primary HBECs isolated from the large and small airways of COPD patients was significantly lower than that in cells from healthy never-smokers and healthy smokers (Fig. 6). The detailed characteristics of these patients are shown in Supplementary Table 1.

## Discussion

The mechanisms of the crosstalk between protease activity and inflammatory processes in HBECs have not been clearly elucidated. This study showed that NE synergistically increases LPS-induced inflammation through PPARγ degradation in HBECs. In accordance with previous studies^17–19^, our study showed that LPS upregulated IL-8 mRNA and protein expression in BEAS-2B cells and primary HBECs; interestingly, our findings also revealed that this upregulated expression was augmented by NE, indicating increased de novo IL-8 protein synthesis. IL-8 is a well-known chemotactic factor that leads to the recruitment of neutrophils, which release NE; this release causes structural damage through extracellular matrix degradation in COPD^20^. The augmentation of LPS-induced IL-8 production by NE suggests that NE potentiates inflammation in addition to directly damaging the tissue. This synergistic interaction could explain the more prominent and perpetual inflammation that occurs when COPD is exacerbated^21^. Although these findings concerning the crosstalk between inflammation and protease activity may not be generalizable, it seems likely that the augmentation is mutual; indeed, the mutual augmentation of IL-8 has also been reported between NE and CS extract (CSE)^13,14^.

Although NE augmented LPS-induced inflammation, the precise mechanism has yet to be clarified. When LPS binds to TLR4, signal transduction is initiated to synthesize IL-8 via the ΙκB/NF-κB pathway^22^; therefore, the first focus was to assess changes in TLR-4 expression. In contrast to the increased TLR4 expression induced by LPS^23^, TLR4 expression decreased following NE administration in total cellular extracts and membrane fractions, which is consistent with a previous report^24^. Next, whether the IκB/NF-κB pathway was involved in the NE-mediated augmentation of LPS-induced IL-8 production was evaluated. In this study, LPS stimulation led to IκBα degradation and the subsequent nuclear translocation of p65 (an NF-κB subunit), as described in a previous report^22^; this effect was not altered by NE treatment. Thus, it seems likely that neither the TLR4 pathway nor the IκB/NF-κB pathway is involved in the NE-mediated augmentation of LPS-induced inflammation. Unlike a previous study, which reported that the one mediated augmentation of CSE-induced IL-8 production was mediated through the extracellular signal-regulated kinase (ERK) pathway^13^, in this study, ERK activation was not involved in the augmenting effect of NE (data not shown), suggesting that the molecular mechanisms might be stimulus-specific.

As neither the TLR4 pathway nor the IκB/NF-κB pathway was involved in the NE-mediated augmentation of LPS-induced IL-8 production, as well as the fact that the increased IL-8 production was due to de novo protein synthesis, we next hypothesized that NE might affect the transcription of IL-8 within the nucleus. Because transcription of IL-8 in the nucleus could be modulated by PPARγ in human lung epithelial cells^16^, the effects of PPARγ were examined. PPARγ is a ligand-activated transcription factor that belongs to the nuclear hormone receptor family. Following translocation into the nucleus, PPARγ interferes with NF-κB and activator protein 1 (AP-1), inhibiting the transcription of proinflammatory cytokines, including IL-8^25^. NE induced the proteolytic degradation of PPARγ. Moreover, the NE-mediated augmentation of LPS-induced IL-8 production was reversed by treatment with either the PPARγ agonist rosiglitazone or overexpression of PPARγ. Taken together, these findings indicate that PPARγ downregulation is responsible for the NE-mediated augmentation of LPS-induced IL-8 production. In accordance with these findings, PPARγ agonists have been shown to attenuate proinflammatory gene expression in the lungs of mice^26^. In addition, PPARγ agonists also ameliorate elastase-induced emphysema in mice^27^. Based on these findings, the PPARγ agonist has been recently spotlighted as a new candidate for COPD treatment^28^.

This study demonstrated that the downregulation of PPARγ resulted in NE-mediated augmentation of LPS-induced IL-8 production in vitro; to extend these findings, the study aimed to determine if these effects translated to in vivo experiments in animals and in human tissues. In accordance with the in vitro findings, the expression of PPARγ was decreased in the lung homogenates of elastase-treated mice. This decrease was most pronounced within bronchial epithelial cells, based on immunohistochemical labeling experiments. Furthermore, it was also found that the PPARγ levels in HBECs obtained from patients with COPD were lower than those in cells from healthy never-smokers or healthy smokers, while current smoking status itself did not affect PPARγ levels (data not shown). Thus, the in vitro and in vivo results were consistent.

Beyond the pathogenesis of stable COPD, the role of PPARγ agonists in COPD exacerbation, which is most frequently caused by respiratory infections, has been suggested. Large population studies from the United States and Taiwan have reported that the use of PPARγ agonists reduced the risk of COPD exacerbation^29,30^; likewise, PPARγ agonists also decreased the risk of exacerbation in patients with asthma^31^. CS-induced neutrophilia followed by nontypeable *Haemophilus influenza* infection was attenuated by rosiglitazone in mice^32^. Although these studies suggest the beneficial effects of PPARγ agonists on COPD exacerbation, the exact mechanisms are still unknown. A future study will focus on investigating the role of PPARγ agonists in a model of COPD exacerbation.

To the best of our knowledge, this is the first study to demonstrate the mechanism by which NE augments LPS-induced IL-8 production in bronchial epithelial cells and to elucidate its role in the pathogenesis of COPD. In conclusion, a vicious cycle of mutual augmentation occurs between protease activity and inflammatory processes through PPARγ degradation; these interactions play a role in the pathogenesis of COPD. These results suggest that the restoration of PPARγ homeostasis could be a new therapeutic strategy in the treatment of COPD.

## Supplementary information

Supplementary information.
