# Supplementary material for: Synergistic cycles of protease activity and inflammation via PPARγ degradation in chronic obstructive pulmonary disease
Source: Exp Mol Med. 2021 May 21;53(5):947–55. doi: 10.1038/s12276-021-00626-7 (PMC8178386; doi:10.1038/s12276-021-00626-7)
Supplement: Supplementary file 1 — Supplementary information. [file 12276_2021_626_MOESM1_ESM.pdf]

## Supplementary Figure 1. NE decreases the expression level of TLR4 protein.

(a) BEAS-2B cells were treated with VC or NE (1 U/mL) for the indicated times. (b) HBECs were treated with VC or NE (1 U/mL) for 24 h. Total RNA was isolated and quantitative real-time PCR for TLR4 and GAPDH was performed (a, b). Data represent the mean $\pm$ SD; ** $P < 0.05$. BEAS-2B cells (c) and HBECs (d) were treated with VC or NE (1 U/mL) for the indicated times. Cell lysates were subjected to western blot analysis for TLR4 and GAPDH quantification. (e) BEAS-2B cells were treated with VC or NE (0.5, 1, 2 U/mL) for 24 h. Membrane fractions were isolated and then subjected to western blot analysis for TLR4 quantification. (f) Cell surface expression of TLR4 was determined by flow cytometry. Results are shown as MFI histograms.

Abbreviations: NE, neutrophil elastase; TLR4, Toll-like receptor 4; VC, vehicle control; HBECs, human bronchial epithelial cells; RNA, ribonucleic acid; PCR, polymerase chain reaction; GAPDH, glyceraldehyde 3-phosphate dehydrogenase; SD, standard deviation; MFI, mean fluorescence intensity.

## Supplementary Figure 1

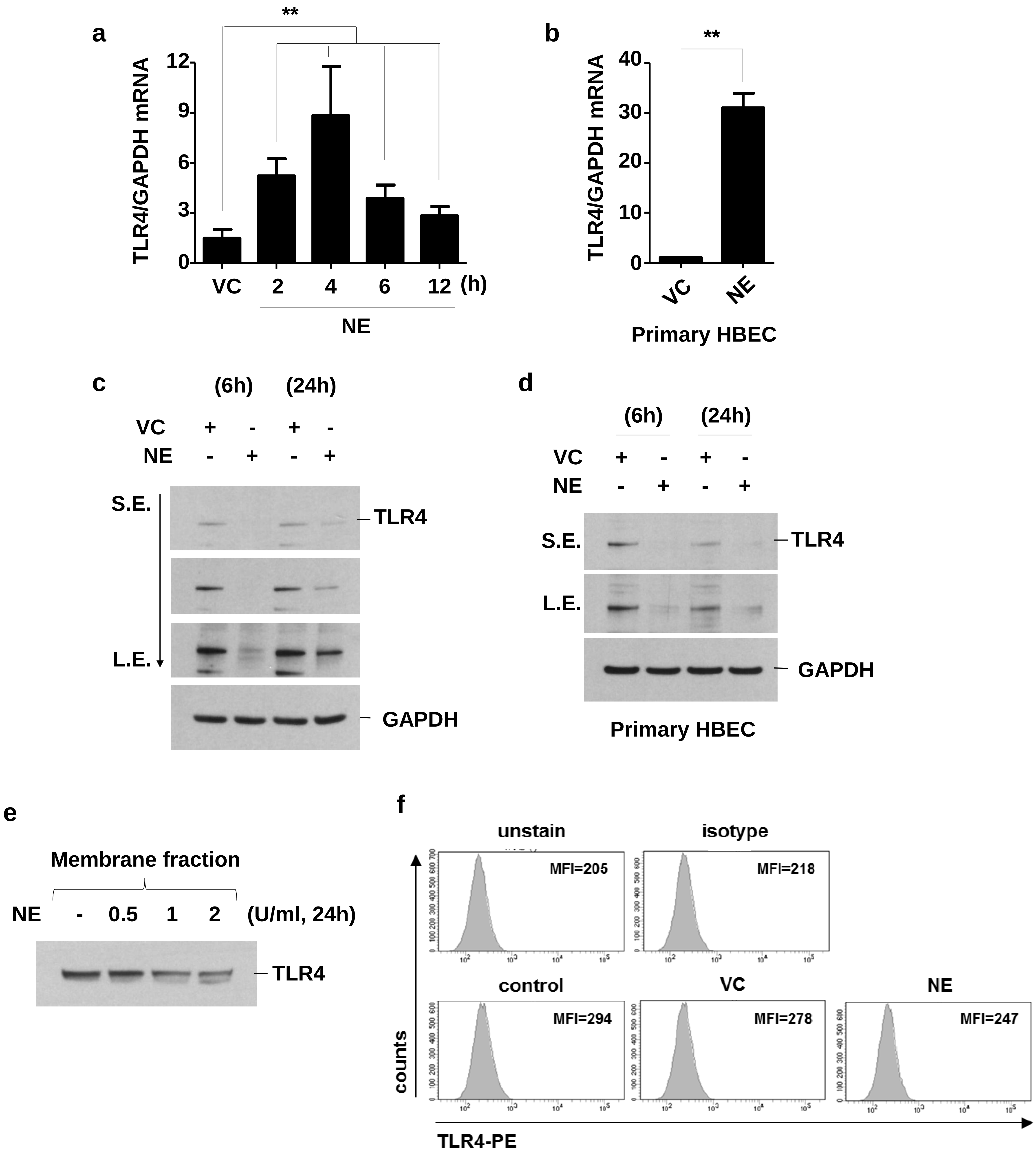

## Supplementary Table 1. Characteristics of patients who provided primary bronchial epithelial cells

| | SAEC | LAEC | Age | Sex | FEV1, Liter (% predicted) | FVC, Liter (% predicted) | FEV1/FVC, % | Smoking status | Pack-years | No. of acute exacerbation during the last year |
|---|---|---|---|---|---|---|---|---|---|---|
| Never smokers | | | | | | | | | | |
| 1 | O | | 65 | Female | 3.06 (145) | 3.65 (126) | 84 | Never | 0 | N/A |
| 2 | O | | 69 | Male | 2.8 (113) | 3.77 (105) | 74 | Never | 0 | N/A |
| 3 | O | O | 62 | Female | 2.16 (113) | 2.77 (105) | 78 | Never | 0 | N/A |
| 4 | O | O | 49 | Female | 2.36 (99) | 2.83 (90) | 83 | Never | 0 | N/A |
| 5 | | O | 74 | Female | 2.11 (130) | 3.02 (128) | 70 | Never | 0 | N/A |
| 6 | | O | 53 | Female | 2.40 (96) | 2.99 (90%) | 80 | Never | 0 | N/A |
| Healthy smokers | | | | | | | | | | |
| 1 | O | O | 75 | Male | 2.83 (119) | 3.16 (88) | 89 | Former | 15 | N/A |
| 2 | O | O | 58 | Male | 2.31 (82) | 3.25 (83) | 71 | Former | 30 | N/A |
| 3 | O | O | 58 | Male | 3.36 (122) | 3.74 (100) | 90 | Former | 10 | N/A |
| 4 | O | O | 62 | Male | 3.39 (127) | 4.46 (120) | 76 | Former | 30 | N/A |
| 5 | O | | 51 | Male | 3.51 (103) | 4.17 (90) | 84 | Former | 30 | N/A |
| 6 | O | | 65 | Male | 3.45 (108) | 4.9 (108) | 70 | Current | 40 | N/A |

| | | | | | | | | | | |
|---|---|---|---|---|---|---|---|---|---|---|
| 7 | O | | 72 | Male | 2.53 (99) | 3.51 (92) | 72 | Former | 60 | N/A |
| 8 | O | | 61 | Male | 2.76 (107) | 3.86 (107) | 72 | Former | 33 | N/A |
| COPD | | | | | | | | | | |
| 1 | O | O | 66 | Male | 0.98 (33) | 3.35 (77) | 29 | Former | 60 | 2 |
| 2 | O | O | 58 | Male | 3.43 (109) | 5.05 (115) | 68 | Former | 50 | 0 |
| 3 | O | O | 74 | Male | 1.05 (42) | 2.79 (74) | 38 | Former | 35 | 0 |
| 4 | O | O | 61 | Male | 2.57 (100) | 4.28 (120) | 60 | Former | 10 | 0 |
| 5 | O | | 64 | Male | 1.87 (71) | 3.45 (93) | 54 | Former | 30 | 0 |
| 6 | O | | 59 | Male | 1.68 (55) | 3.9 (91) | 43 | Current | 40 | 0 |
| 7 | O | | 83 | Male | 1.18 (70) | 2.15 (79) | 55 | Former | 75 | 1 |
| 8 | O | | 61 | Male | 1.15 (39) | 3.60 (86) | 32 | Former | 40 | 1 |
| 9 | O | | 64 | Male | 1.59 (59) | 3.43 (91) | 46 | Current | 30 | 0 |
| 10 | O | | 74 | Male | 1.34 (60) | 2.80 (84) | 48 | Former | 44 | 0 |
| 11 | O | | 76 | Male | 1.76 (76) | 3.60 (102) | 49 | Former | 39 | 0 |

SAEC, small airway epithelial cells; LAEC, large airway epithelial cells; FEV1, forced expiratory volume in 1 second; FVC, forced vital capacity; COPD, chronic obstructive pulmonary disease; N/A, not-applicable.
